# Simultaneous Full‐Color Printing and Holography Enabled by Centimeter‐Scale Plasmonic Metasurfaces

**DOI:** 10.1002/advs.201903156

**Published:** 2020-03-16

**Authors:** Fei Zhang, Mingbo Pu, Ping Gao, Jinjin Jin, Xiong Li, Yinghui Guo, Xiaoliang Ma, Jun Luo, Honglin Yu, Xiangang Luo

**Affiliations:** ^1^ State Key Laboratory of Optical Technologies on Nano‐Fabrication and Micro‐Engineering Institute of Optics and Electronics Chinese Academy of Sciences Chengdu 610209 China; ^2^ Key Laboratory of Optoelectronic Technology and System Ministry of Education Chongqing University Chongqing 400030 China; ^3^ School of Optoelectronics University of Chinese Academy of Sciences Beijing 100049 China

**Keywords:** holograms, metasurfaces, plasmonics, structural colors

## Abstract

Optical metasurfaces enable novel ways to locally manipulate light's amplitude, phase, and polarization, underpinning a newly viable technology for applications, such as high‐density optical storage, holography, and displays. Here, a high‐security‐level platform enabled by centimeter‐scale plasmonic metasurfaces with full‐color, high‐purity, and enhanced‐information‐capacity properties is proposed. Multiple types of independent information can be embedded into a single metamark using full parameters of light, including amplitude, phase, and polarization. Under incoherent white light, the metamark appears as a polarization‐ and angle‐encoded full‐color image with flexibly controlled hue, saturation, and brightness, while switching to multiwavelength holograms under coherent laser illumination. More importantly, for actual applications, the extremely shallow functional layer makes such centimeter‐scale plasmonic metamarks suitable for cost‐effective mass production processes. Considering these superior performances of the presented multifunctional plasmonic metasurfaces, this work may find wide applications in anticounterfeiting, information security, high‐density optical storage, and so forth.

## Graphical abstract

Both full‐color printing and holography can be realized by a single ultrathin plasmonic metasurface. Under incoherent white light, the proposed centimeter‐scale metasurface appears as a polarization‐ and angle‐encoded full‐color image with flexibly controlled hue, saturation, and brightness, while switching to multiwavelength holograms under coherent laser illumination. More importantly, such metamarks allow large‐area and mass production at low cost.

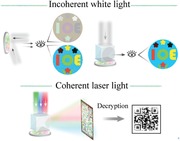

## 1 Introduction

With the advance in nanofabrication technology, a considerable interest has been attracted to the emerging field of artificially structured materials, which enable spatial control of electromagnetic waves.^1^, ^2^ As their 2D counterparts, metasurfaces consisting of subwavelength structures enable flexible manipulation of light's fundamental properties like amplitude, phase, and polarization, by properly utilizing strong interactions between subwavelength structures and electromagnetic waves.^2^, ^3^, ^4^ Many exotic phenomena and extraordinary planar optical devices have been realized, including photonic spin–orbit interactions (SOIs),^5^, ^6^, ^7^ spatiotemporal light control,^8^ ultrafast optical pulse shaping,^9^ achromatic wavefront shaping,^10^, ^11^ invisibility cloaking,^12^ full‐color 3D holography,^13^, ^14^, ^15^ structural color printing,^16^, ^17^, ^18^ and many others.^19^, ^20^, ^21^, ^22^ Among the plethora of applications currently attracting great interests, both metaholograms and metaprints are promising as the next‐generation optical security/storage devices.^23^, ^24^ Generally, metaholograms typically tailor the phase, whereas metaprints modulate the amplitude of light.

To enhance/strengthen the information capacity/security, simultaneous control of the phase and amplitude has been proposed to achieve the integration of printing and holography by a single metamark.^25^, ^26^, ^27^, ^28^, ^29^, ^30^ The metamark appears as a color image under white light, while operating as the hologram under laser illumination. Despite the demonstrations of the dual‐mode display by these preliminary works,^25^, ^26^, ^27^, ^28^, ^29^, ^30^ it is still of an extreme challenge to simultaneously obtain full‐color printing and holography with large scale, impeded by the fundamental limitations and fabrication unfriendliness as below. Table S1 (Supporting Information) provides an overview of these relevant works on the dual‐mode display. Generally, such a function requires diverse meta‐atoms with separated reflection/transmission peaks, for example, at red, green, and blue wavelengths. Well‐separated property enables not only low crosstalk among multiple hologram channels but also high‐purity color generation in the print mode. Many efforts have been paid on sharp spectrum generation by selectively enhancing/suppressing the amplitudes at resonant/off‐resonant wavelengths, and the spectral bandwidth usually depends on the strength of the resonance.^17^, ^31^ How to encode phase shifts at the resonant wavelength is still challenging, because the peak wavelength usually undergoes the red‐ or blueshift if the phase shift is introduced by changing the meta‐atom size. The spectral shift will cause information distortion and noise enhancement. To solve this issue, 3D‐integrated transmission‐type metamaterials composed of two functional layers (one acts as color filters and another introduces phase shifts) have been proposed to achieve decoupled phase and amplitude control,^28^, ^29^ but with the cost of time‐consuming/expensive 3D fabrication and still suffering from limited colors. Furthermore, other reported dual‐mode metasurfaces,^25^, ^26^, ^27^, ^30^ which depend on point‐by‐point scanning methods like electronic beam lithography or external complex procedures, still suffer from extremely poor production efficiency, which prevents their scalable applications from postprocessing customization.

Here, we present ultrathin plasmonic metasurfaces for simultaneous full‐color printing and holography with centimeter‐scale, mass‐production, and low‐cost characteristics. Simultaneous amplitude and phase control are realized by photonic spin–orbit interactions that are excited by surface plasmon polariton (SPP) within a narrow spectral band. The narrowband response contributes to not only high‐purity color generation but also low crosstalk among hologram channels. A full‐color image and three holographic images can be encoded onto a single metamark in its print and hologram modes, respectively. Furthermore, owing to the ultrahigh‐purity color generation by the proposed plasmonic structures, the hue, saturation, and brightness of the printing image can be flexibly controlled through the color mixing theory, and the printing image shows diverse colors under different observation conditions. Those properties make it applicable to multilevel security identification, optical storage, and so forth. More importantly, its extremely shallow depth is suitable for the surface plasmon (SP) lithography and coating process, both of which belong to cost‐effective, mass‐production, large‐area fabrication processes.^32^, ^33^

## 2 Results

### 2.1 The Concept of Multilevel Security Identification

**Figure 1:**
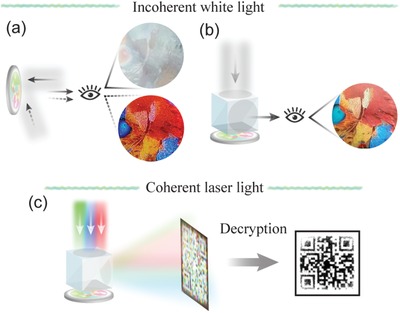
Operation schematic of the proposed plasmonic metasurface for multilevel security identification. a) First, a tine image and a colorful image are observed in the reflection and diffraction modes, respectively, under incoherent white light. b) Second, the metasurface reveals a colorful image in the reflection mode with the decryption device. c) Third, the metasurface works as multiwavelength holograms when illuminated by coherent laser light. The hidden information (QR code) can be decoded from three holographic images merely at right wavelengths using a specific algorithm. Bird image adapted with permission. Copyright PiperAnne Worcester/DanitaDelimont.com.

**Figure** **1** schematically illustrates the proposed plasmonic metasurfaces applied to multilevel security identification. When directly viewed by the naked eyes in incoherent white light (e.g., the light from a lamp or sun), the metamark appears as a tint image in the reflection mode at normal incidence but changes to a colorful one in the diffraction mode at near‐glance incidence, as shown in Figure 1a, which is regarded as the first level of security validation owing to its detection simplicity. To prevent being faked by traditional cheap counterparts, a decryption device consisting of two linear polarizers, one beam splitter, and one quarter waveplate is employed to carry out the second level of security checking using the polarization degree of freedom, and then the information hidden in the cross‐polarized component can be decoded. As shown in Figure 1b, the metamark reveals a colorful image in the reflection mode at normal incidence (Figure 1b). After passing the first two simple checks, the final one requires tricolor (red, green, and blue) coherent laser illumination to project a synthetically color holographic image onto a screen placed in the far field (Fraunhofer regime), as sketched in Figure 1c. Furthermore, a specific algorithm is required to complete information decryption from the diffraction patterns.

### 2.2 Principle

As a wise way to implement phase control while simultaneously maintaining the structural color, wavelength‐independent geometric phase (also refers to Pancharatnam–Berry phase), which is carried on the polarization‐converted circularly polarized (CP) light and originates from photonic spin–orbit interactions,^34^ can be introduced by rotating the meta‐atom. Assuming a meta‐atom with an orientation of α, the reflected field from the meta‐atom by CP light (+σ) at normal incidence and observation can be written as^35^

(1) $$E_{\text{r}}|\sigma 〉=\frac{r_{\text{l}}+r_{\text{s}}}{2}|\sigma 〉+\frac{r_{\text{l}}-r_{\text{s}}}{2}e^{-j2\sigma \alpha }|-\sigma 〉$$

where σ is selected to be +1 or −1 for right‐ or left‐handed CP light; *r*_l_ and *r*_s_ are the complex reflection coefficients for linearly polarized light along the fast and slow axes of the meta‐atom. The phase factor of −2*σα* in the right term of Equation (1) is the free‐dispersion geometric phase that carries on the cross‐polarized CP reflection component. By arranging rotated meta‐atoms, an arbitrary phase distribution can be obtained to code a desired holographic image, while hardly changing their amplitude spectra, that is, structural colors. As the geometric phase exists once the polarization conversion occurs, it is required that polarization conversion happens within a narrow spectral band to avoid crosstalk among multiple hologram channels. It means that there needs to be a sharp propagation phase difference of π between the fast and slow axes (assuming no losses) around the peak wavelength. Furthermore, the high sharpness can also contribute to high‐purity color generation for the cross‐polarized component. By adjusting the size of the meta‐atom, the peak wavelength could be shifted toward either higher or lower wavelengths in theory,^25^ resulting in various structural colors and multiwavelength holograms.

The fact that the high‐purity color is carried on the cross‐polarized component can add an extra degree of freedom for information encryption, but it also requires the decryption device to generate broadband CP light and filter the copolarized component. By proper design shown below, similar colors can be observed in the diffraction mode without any external devices. Assuming that a 1D or 2D meta‐atom array has the period of *p* along the *x*‐direction and is obliquely illuminated at an angle of θ_i_ along the *x*‐direction, the central wavelength of the 1st‐order diffraction component at an observation angle of θ_o_ along the *x*‐direction can be expressed as

(2) $$\lambda =p\times (sin\theta_{\text{i}}-sin\theta_{0})$$

where *p* indicates the period of the meta‐atom; θ_i_/θ_o_ represents the angle between the normal line and incident/observation direction. The various colors can be generated by the meta‐atom array with different periods for the same illumination and observation angles. Conversely, the central wavelength (i.e., the observed color) for a fixed period depends on both ones. Importantly, if captured by a small aperture one, like the naked eye or a smartphone camera, the collected spectral bandwidth can be written approximately as

(3) Δλ  =  ap/d

where *a* indicates the aperture of the eye or camera and *d* is the observation distance. Generally, *d* ≫ *a*, which indicates that the observed colors should be very pure. To avoid image distortion, the observation angle refers to be close to 0°, so that the incident angle should be close to 90°.

### 2.3 Structure Design

**Figure 2:**
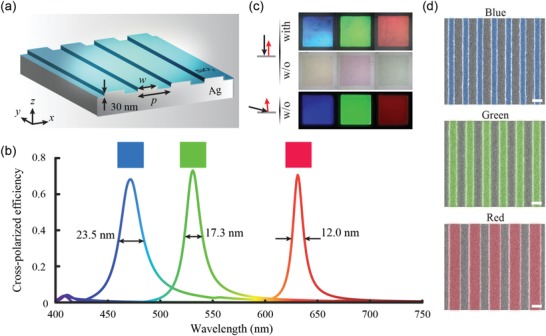
Demonstration of high‐purity color generation. a) The schematic illustration of proposed PSG, showing an extreme low structural height of 30 nm. b) Simulated cross‐polarized spectra of three different PSGs. Corresponding periods and widths are as followed: *p* = 414.3 nm, *w* = 185 nm (blue); *p* = 483.3 nm, *w* = 240 nm (green); *p* = 580 nm, *w* = 380 nm (red). Insets: calculated colors under D65 illuminated light. c) Optical images of fabricated PSGs in the reflection and diffraction modes. “with” means the use of the decryption device; “w/o” means no use of the decryption device. d) SEM images of fabricated PSGs. Scale bar: 300 nm. Highlight regions are bulged.

For traditional single‐layer and ultrathin metallic rods or slits, there is almost no propagation phase difference between the fast and slow axes, resulting in low cross‐polarized efficiencies and broadband cross‐polarized spectra.^13^, ^14^ However, such a requirement of the sharp phase difference can be satisfied by our reported silver (Ag) plasmonic shallow gratings (PSGs).^36^ The proposed PSG here shown in **Figure** **2**a has a groove depth of 30 nm with a thin (3 nm) silicon dioxide (SiO_2_) layer on the Ag surface to prevent it from oxidation and sulfation in ambient air. Such a shallow depth allows the PSG to be manufactured through merely one single‐step SP lithography and several coating processes (see the Experimental Section for details). Ag is chosen because it enables strong plasmonic responses at visible wavelengths. The catenary optical field exited by SPP, which has been found to play an important role in subwavelength electromagnetics,^37^ can introduce an abrupt phase shift for the *x*‐polarization incidence, while it does not happen for the *y*‐polarization incidence. As a result, there is a sharp propagation phase difference of π between the fast and slow axes at the resonant wavelength, further leading to narrowband photonic spin–orbit interactions. See Section S1 of the Supporting Information for details. The resonant wavelength of SPP can be determined by the relation^36^, ^38^

(4) $$\lambda_{\text{SPP}}=\xi (w)p\sqrt{\frac{\varepsilon_{\text{m}}\varepsilon_{\text{d}}(t)}{\varepsilon_{\text{m}}+\varepsilon_{\text{d}}(t)}}$$

where λ_SPP_ is the vacuum wavelength; ξ(*w*) is the correction factor and indicates that λ_SPP_ can also be tuned by the duty cycle of the PSG; *p* represents the period of the PSG and equals to the effective wavelength for SPP; ε_m_ is the dielectric function of the silver; ε_d_(*t*) is related to the thickness of SiO_2_ layer and represents the effective dielectric function of the combination of the thick SiO_2_ layer and air. According to Equation (4), the peak wavelength depends not only on the thickness of the protective layer but also the period and width of the PSG. Thus, the demand for other forms of protective layers can also be satisfied by optimizing the width and period.

Considering the aforementioned factors, three PSGs corresponding to the peak wavelengths of 473, 532, and 633 nm, respectively, are designed with their geometrical parameters given in the figure caption. Figure 2b shows their cross‐polarized reflective spectra under the normal illumination of CP light. Reflective efficiency peaks reach over 70%, and the full width at half magnitude (FWHM) values are ≈23.5, 17.3, and 12 nm for the blue, green, and red PSGs, respectively. The mechanism that the red PSG shows a smaller FWHM value is analyzed in Section S1 of the Supporting Information. The high resonance sharpness and low spectral background contribute to not only high‐purity color generation but also low‐crosstalk multiwavelength holograms (a high signal‐to‐noise ratio of >17:1 among three peek wavelengths is supported). Such a signal‐to‐noise ratio is lower than that of reported off‐axis metaholographic techniques,^13^ but it is higher than that of previous relevant works on simultaneous color printing and holography (see Table S1, Supporting Information). Figure 2c sketches their optical microscope images using the halogen lamp source, and their scanning electron microscopy (SEM) images are sketched in Figure 2d. With the decryption device, three PSGs display high‐purity blue, green, and red, as shown in the top row of Figure 2c. Measured results agree well with the simulations using the D65 white source (insets of Figure 2b). The possible reasons for the little discrepancy include the fact that the simulations assume absolute cross‐polarized component, whereas copolarized one exists in the experiments owing to two factors, including the limited bandwidth performance of the elements in the decryption device (especially, the quarter waveplate) and the ghost caused by multiple reflections among optical elements (Figure S3, Supporting Information). Besides, fabrication imperfections and none‐normal illumination from a microscope objective lens might contribute (Figure S4, Supporting Information). The simulation results in Figure S5 (Supporting Information) show that a fabrication tolerance of ±15 nm in the lateral size is still acceptable to yield reasonable performance. As shown in the middle panel of Figure 2c, these PSGs display three similar‐complementary colors without the decryption device, owing to some absorption around the resonant wavelengths (Figure S6, Supporting Information). According to Equations (2) and (3), when the positions of the light source and small‐aperture camera are fixed, the colors of PSGs contributed by the 1st‐order diffraction light change gradually with the rotation of samples (see Figure S7 for details, Supporting Information). When rotated to a suitable angle, the observed colors in the diffraction mode shown in the bottom of Figure 2c are very similar to ones produced by the cross‐polarized component in the reflection mode. These properties can be employed to implement the first two levels of security identification shown in Figure 1a,b.

### 2.4 Integration Design of Full‐Color Printing and Holography

Merely tricolor display rarely encodes complex image information that is of critical importance for many practical applications. By exploiting meta‐atoms with different sizes, various colors can be readily obtained. In this work, we specialize in dual‐functional metamarks—that is, displaying more colors in the print mode will, in turn, cause pixel loss and noise enhancement in the hologram mode. Moreover, the energy efficiency in the hologram mode is also influenced by the number of the meta‐atoms with their peak wavelengths to match the corresponding hologram channel, so the efficiency will be greatly reduced if the printing image has rich colors if relying on merely structural geometries.^28^

**Figure 3:**
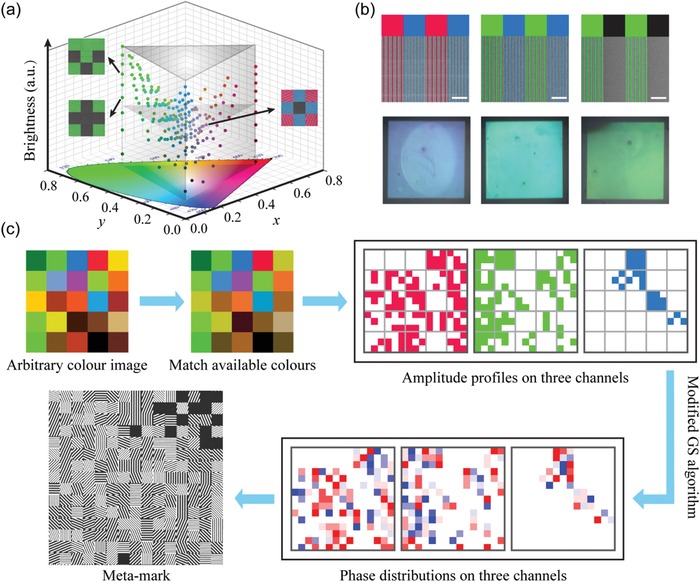
Working principle of simultaneous full‐color printing and multichannel holography. a) Schematic illustration of color adjustment using the supercell combined with phase control ability. b) Experimental demonstrations of color mixing. Top row: SEM images (scale bar: 2 µm). Bottom row: optical microscope images. c) Schematic diagram of designing a metamark for simultaneous full‐color printing and holography.

Owing to the ultrahigh purity of three primary colors generated by the presented PSGs and according to color mixing theory, the colors containing in the triangle (≈38.1% of the whole International Commission on Illumination (CIE) 1931 chromaticity diagram) shown in **Figure** **3**a can be obtained with flexible brightness tunability when three primary colors overlap in a proper mixture, which can be implemented by the supercell. Here, to simultaneously implement phase control, the rotated PSG is pixelated with a size of 2.9 µm × 2.9 µm, which corresponds to ≈4.6 times maximum peak wavelength (633 nm), and then arranged as the form of *N* × *N* array to construct a supercell. Each supercell contains from zero to *N* ^2^ pixelated PSGs. Each subpixel consists of five, six, and seven rods for three sorts of pixelated PSGs. If the demand for a higher resolution is crucial, each subpixel can contain less rods, but at the cost of a lower efficiency.^39^ When observed with the decryption device, red, green, blue, and black can be mixed in a supercell with an adjustable ratio, and thus each supercell represents a pixel of an additive synthetic color. The number of additive colors can be written as

(5) $$\text{num}=\sum_{i=1}^{N^{2}+1}\sum_{j=1}^{i}j$$

Here, we employ 3 × 3 array, which corresponds to ≈13.7 times maximum peak wavelength (633 nm), with an image resolution of ≈2920 ppi, and 220 additive colors can be obtained. As shown in Figure 3a, this pixel scheme is readily capable of creating abundant additive colors with hue, saturation, and brightness tunability. Furthermore, by adding more subpixels in a supercell, for example, 3276 additive colors can be obtained for the form of 5 × 5 array, but it has no contribution to increasing the color gamut. Owing the ultranarrowband spectral characteristics of the proposed PSGs, five different PSGs can also be designed within 400–750 nm (Figure S8, Supporting Information), while maintaining a high signal‐to‐noise ratio of ≈10:1 among nine peek wavelengths. In this case, a larger color gamut (≈49.2%) can be obtained and five‐wavelength holograms can be realized theoretically. Figure 3b shows the SEM and optical microscope images of three samples with the form of 3 × 3 array for proof‐of‐concept demonstrations. As predicted, an addictive color can be obtained by mixing different PSGs in a supercell, and the brightness is also successfully tuned by the ratio between pixelated PSGs and Ag mirror, demonstrating the capability for full‐color printing.

Subsequently, as shown in Figure 3c, we present the design strategy for simultaneous full‐color printing and multichannel holography. First, each pixel of an arbitrary color image to be patterned is color‐matched to the closest available additive colors, and the combination of three sorts of pixelated PSGs is subsequently determined. The PSG subpixels in each supercell are, however, randomly arranged to suppress the high‐order diffraction noises in the hologram mode caused by the unwanted periodicity for regular arrangement (Figure S9, Supporting Information). After obtaining the amplitude profiles on three hologram channels, modified Gerchberg–Saxton algorithm (see Section S3.3 of the Supporting Information) is applied to calculate corresponding phase profiles at three peak wavelengths of 473, 532, and 633 nm (we call them blue, green, and red laser light hereafter), respectively. As a result, a large color gamut (≈62.5% of the whole CIE 1931 chromaticity diagram) can be obtained in the hologram mode. Finally, the desired metamark can be constructed according to the amplitude and phase profiles with corresponding PSG subpixels.

Compared with the previous color metaholograms using surface plasmonic resonances,^14^, ^15^ such a metamark enables not only high‐efficiency multichannel holography but also full‐color printing, thus this integration design could further enhance the larger information capacity. Although the pixel density of the proposed metamark is about one thirteenth of that of commercial blue disks, it has more degrees of freedom like wavelength, amplitude (color), phase, and polarization to code information, holding attractive opportunities to further enhance the information capacity and security. Besides, to further strengthen the security of the metamark and prevent it from the reproduction through its SEM images, a thick (175 nm) covering film can be designed to hide the structure, as shown in Figure S10 (Supporting Information).

### 2.5 Experimental Demonstrations of Simultaneous Full‐Color Printing and Holography

**Figure 4:**
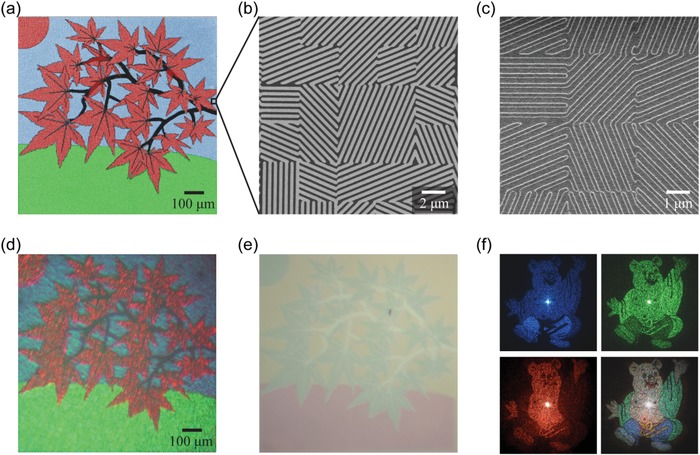
Demonstration of simultaneous amplitude and phase control. a) Arrangement of PSGs for a tricolor print with red, green, and blue regions corresponding to three sorts of PSGs, respectively. b) SEM image of fabricated Pr patterns, showing phase control by pixelated rotated PSGs. c) Enlarged SEM image after depositing Ag and SiO_2_ on the Pr patterns. Two optical microscope images of the tricolor metamark d,e) with/without the decryption device, respectively. f) Measured far‐field holographic images in the hologram mode of the tricolor metamark under the coherent illumination of only blue (top left), green (top right), red (bottom left), and all three lasers simultaneously (bottom right).

As a proof‐of‐concept demonstration of simultaneous amplitude and phase control, we designed, fabricated, and characterized a tricolor metamark (size: 1 mm × 1 mm) that encodes a landscape in the print mode and three independent images of cartoon pigs in the hologram mode, respectively. As shown in **Figure** **4**a–c, red, green, and blue pixelated PSGs with different orientations are arranged in the leave, lawn, and sky regions, respectively, to form a tricolor printing image, and each array contributes to only one hologram channel. Under incoherent white light, the phase modulation of the holograms is effectively ignored and the rotated PSGs act as only amplitude‐modulating color pixels that together show the desired printing image. Figure 4d,e compares its two optical microscope images using the halogen lamp source with/without the decryption device, showing a good agreement with the colors performed by the PSGs (Figure 2c). Little noise can be attributed to the factors aforementioned in the last paragraph of Section 2.3. When illuminated by the coherent laser light, the metamark will be switched from the print mode into the hologram mode, resulting in the appearance of holographic images in the Fraunhofer regime. Shown in Figure 4f are measured diffraction patterns under the normal illumination of blue, green, and red laser light, respectively. Each hologram channel can generate an independent diffraction pattern with neglectable crosstalk from others. Hence, a high‐quality color cartoon pig is obtained when illuminated by synthetically tricolor laser light (the bottom‐right panel in Figure 4f). Since each array contributes only one hologram channel, the total efficiency defined as the sum of the efficiencies of three hologram channels was characterized, which should in theory be close to the average polarization‐conversion efficiency of three PSGs at their peak wavelengths, if there is no black area in the printing image. The measured total efficiency is ≈48.2%, which is lower than the simulated average efficiency. The reason is that about 10% of the area (branches) is black and has no contribution to the holograms. Besides, fabrication errors may also contribute to this discrepancy.

**Figure 5:**
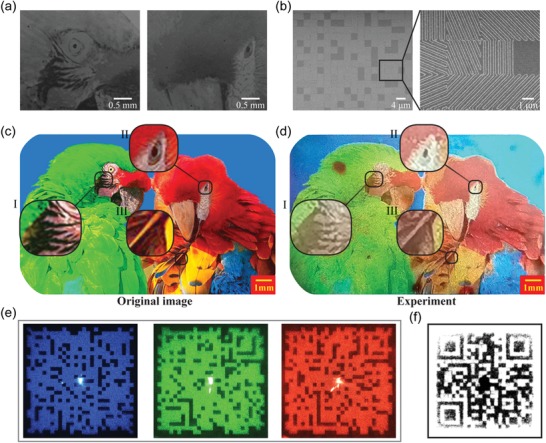
Demonstration of simultaneous full‐color printing and multichannel holography. SEM images of a) Pr patterns and b) complete structures after depositing Ag and SiO_2_. c) Original image of two macaws. d) An optical image of the fabricated macaw metamark with the decryption device. Bird image adapted with permission. Copyright PiperAnne Worcester/DanitaDelimont.com. e) Measured holographic images under the coherent illumination of blue, green, and red laser light. f) Decrypted QR code from three measured holographic images.

For the further demonstration of full‐color capability and large‐area production, a centimeter‐scale metamark with a size of 10 mm × 16 mm was fabricated with its SEM images sketched in **Figure** **5**a,b. A colorful image of two macaws was chosen as the printing image, and three modified QR codes are embedded in its hologram mode to demonstrate holographic encryption shown in Figure 1c. Owing to the ultralarge scale, its optical images were captured by the normal smartphone camera, which means that the portability and internet‐accessibility of smartphones can be applied for real‐time trace, track, and verification of genuine information. Furthermore, such a large scale also enables the observation by the naked eyes, making the preliminary verification simpler. Figure 5c,d compares the original image and an optical image of the macaw metamark formed by the smartphone camera with the decryption device using the xenon light source. Benefiting from the large color gamut supported by the proposed supercells, the observed image is consistent with the original one, except for tiny defects and little discrepancy caused by fabrication errors. Furthermore, the ghost caused by multiple reflections within the decryption device may also contribute to the discrepancy in the top‐right area, however, which can be avoided by coating antireflection layers on the optical element surfaces. As shown in Figure 5d, apart from red, green, and blue, other synthetic colors with brightness gradient like orange, yellow, and magenta are also observed, which unambiguously demonstrates the color mixing capability of the proposed approach. Without the decryption device, these two macaws also display their similar‐complementary colors (Figure S11, Supporting Information). Besides, a colorful image similar to the one sketched in Figure 5d can be observed in the diffraction mode (Figure S12, Supporting Information).

Under the illumination of coherent laser light, the modified QR code will appear in the Fraunhofer region as we predicted. Figure 5d depicts three measured diffraction patterns when illuminated by blue, green, and red laser light. It can be seen that they are very clear, demonstrating neglectable crosstalk among three hologram channels. In special, a decryption algorithm is designed together with the holograms, so that the real QR code can be decoded from these holographic images with three right wavelengths (see Figure S13 for details, Supporting Information). Then, the encrypted information in the QR code shown in Figure 5f can be directly read through the smartphone. We measured ≈41.9% total efficiency of three hologram channels, which is lower than that of the tricolor one owing to the brightness control of the printing image. Since the input color printing image has a varied brightness distribution, the reflection amplitude through the metasurface cannot reach the maximal values at all positions, therefore limiting the optical efficiency in the hologram mode. If one is interested only in the hologram functionality, the theoretical limit of the optical efficiency is ≈70% according to Figure 2b. Owing to the large‐area property, spatial multiplexing technology (different regions of the metamark contribute to different holographic images even for the same wavelength) can be employed to further enhance information capacity and decryption difficulty. Since the information capacity is fixed at a given size of the metamark, the more channels, the smaller the corresponding information capacity of each channel, and the lower the corresponding quality in each channel. The degree of quality degradation depends on the complexity of the encoded information, that is, the simpler the encoding information, the more independent channels can be allowed.

## 3 Conclusion

In conclusion, the proposed ultrathin plasmonic metasurfaces enable the mode switching between full‐color printing and multichannel holography by the coherence of incident light. Under broadband incoherent light, the colors of the printing image vary with both illumination and observation angles, and it can be switched from the pseudo to the actual using the polarization degree of freedom. Meanwhile, it can serve as low‐crosstalk multiwavelength holograms when illuminated by coherent laser light. More importantly, the proposed plasmonic metasurfaces may allow large‐area and mass production at low cost, owing to the fabrication simplicity. These superior properties provide a promising route for anticounterfeiting technology. Increasingly rampant counterfeiting activities force the growth of the global anticounterfeiting market with a compound annual growth rate of 11.7% in recent years, and by 2023 the global anticounterfeiting packaging market is predicted to reach 208.4 billion USD.^40^ Compared with some conventional technologies, the proposed plasmonic metasurface has more degrees of freedom to encode complex information, because the amplitude, phase, and polarization of light are simultaneously employed as information carriers during the design of the proposed metamarks. The unique combination of high security, friendliness for fabrication, and cheap mass production makes this design an easy way to bridge the gap between theoretical investigations and daily‐life applications.

In the future studies, the ultrathin property may allow the proposed metamarks to be fabricated on flexible substrates and then to be transformed on a curved surface for more applications. Furthermore, low‐cost and large‐scale metamarks presented here could be shared with smartphone‐based security identification technologies. Besides, its ultranarrowband wavefront manipulation ability is promising for the applications of multispectral imaging, spectral retrieval,^41^ and so forth. In addition, the microscale grating arrays can also be designed as micropolarizers for polarimetric imaging. We believe that this research may provide many new ideas for subwavelength electromagnetics,^42^, ^43^ allowing us to envision applications in display applications, information security engineering, biomedical sensing, and more.

## 4 Experimental Section

##### Simulations

To better conform to the actual situation, the unit cell in the simulation is schematically sketched in Figure S14 (Supporting Information) according to the fabrication procedures. The simulation results for the unit cell in this paper were obtained by CST Microwave Studio based on the finite element method. The unit‐cell boundary condition was applied at both *x*‐ and *y*‐directions. For the *z*‐direction, the open boundary condition was employed. The dielectric function of Ag was obtained from the data measured by Johnson and Christy,^44^ the permittivities of SiO_2_ and photoresist were obtained from the measurement (Figures S15 and S16, Supporting Information), and the permittivities of magnesium fluoride were obtained from the Palik′s handbook.^45^

##### Sample Fabrications

**Figure 6:**
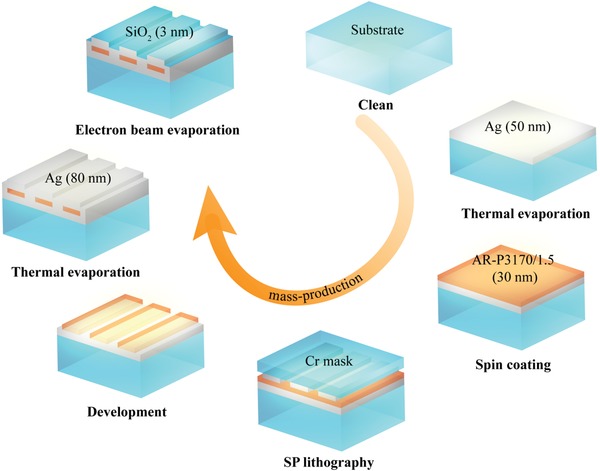
The fabrication process of the proposed centimeter‐scale plasmonic metasurfaces. This technique can enable mass, large‐area, and low‐cost production, which is promising in industrial applications.

The fabrication process is schematically shown in **Figure** **6**. A 50 nm thick Ag thin film was deposited on a SiO_2_ substrate by thermal evaporation. Then, the photoresist (Pr, AR‐P3170/1.5) with a thickness of 30 nm was spin‐coated on the sample. After that, a SP lithography, which is one kind of super‐resolution optical lithography with the minimum resolution being 22 nm,^46^ was employed to transfer patterns on a reusable Cr mask fabricated by electronic beam lithography to the Pr film. After development, an Ag film with a thickness of 80 nm was deposited on Pr patterns using thermal evaporation. Finally, a 3 nm thick SiO_2_ film was subsequently deposited through electron beam evaporation. All employed processes enabled large‐area and batch production at low cost. The coating process could be applied to dozens or even hundreds of the proposed centimeter‐scale plasmonic metasurfaces at the same time. Therefore, as for mass production, the fabrication efficiency mainly depended on the time of lithography procedure. The SP lithography technology does not belong to point‐by‐point scanning methods, and one single‐step lithography usually requires several seconds. Considering the time to install the sample, adjust the system, expose the Pr film, and remove the sample, one single lithography procedure totally requires ≈90 s. For the production line in future, the time except exposure could be greatly compressed. As a result, it was thought that the proposed centimeter‐scale metasurfaces may allow mass production at low cost.

##### Characterizations

Shown in Figure S17a (Supporting Information) is the schematic diagram of the optical setup for the hologram characterizations. A tunable supercontinuum laser source (NKT‐SuperK EXTREME), which can simultaneously output multiwavelength light beams, was employed as the coherent light source. An achromatic Fourier lens was applied to perform optical Fourier transformation, and the holographic images were captured by a charge‐coupled device (CCD). For the efficiency characterizations, the CCD was replaced by a power meter. As illustrated in Figure S17b (Supporting Information), the optical images of the small‐area samples, including the landscape metamark and several PSGs, were captured by a microscope with a × 4/0.1 objective lens. The optical setup for the macaw metamark is displayed in Figure S17c (Supporting Information). It is noteworthy that the decryption device was replaced by a single beam splitter when characterizing their corresponding pseudoimages in the reflection mode. Furthermore, their optical images in the diffraction mode were captured through the optical setup shown in Figure S7a (Supporting Information).

## Conflict of Interest

The authors declare no conflict of interest.

## Supporting information

Supporting Information
