# Supplementary material for: Simultaneous Full‐Color Printing and Holography Enabled by Centimeter‐Scale Plasmonic Metasurfaces
Source: Adv Sci (Weinh). 2020 Mar 16;7(10):1903156. doi: 10.1002/advs.201903156 (PMC7237853; doi:10.1002/advs.201903156)
Supplement: Supplementary file 1 — Supporting Information [file ADVS-7-1903156-s001.pdf]

Copyright WILEY-VCH Verlag GmbH & Co. KGaA, 69469 Weinheim, Germany, 2020.

ADVANCED SCIENCE
Open Access

Supporting Information

for *Adv. Sci.,* DOI: 10.1002/advs.201903156

Simultaneous Full-Color Printing and Holography Enabled by Centimeter-Scale Plasmonic Metasurfaces

*Fei Zhang, Mingbo Pu, Ping Gao, Jinjin Jin, Xiong Li, Yinghui Guo, Xiaoliang Ma, Jun Luo, Honglin Yu, and Xiangang Luo**

Supporting Information

Simultaneous full-color printing and holography enabled by centimeter-scale plasmonic metasurfaces

*Fei Zhang, Mingbo Pu, Ping Gao, Jinjin Jin, Xiong Li, Yinghui Guo, Xiaoliang Ma, Jun Luo, Honglin Yu and Xiangang Luo**

1. The mechanism of the sharp phase difference

To explore the physical mechanism inside narrowband phonic spin-orbit interactions, the electric-field distributions of the blue PSG at different wavelengths are simulated under the illumination of the *x*- and *y*-polarizations, respectively, as shown in Figure S1a,b. The top row of Figure S1a,b show the amplitude distributions of E_x and E_y that are normalized to the incident magnitude ($|E_i|$), and the bottom row displays the corresponding electric-field lines. As depicted in Figure S1a, the electric field in obviously enhanced when under the illumination of the *x*-polarization incidence, but it does not occur for the *y*-polarization incidence. For the *x*-polarization incidence, the electric field is strongly coupled between the two corners of an element at the wavelength of 450 nm, as shown in Figure S1(i). In contrast, Figure S1(iii) shows that the electric field is reinforced between two adjacent elements at the wavelength of 500 nm. When at the resonant wavelength of 473 nm, those two modes are strongly coupled, as illustrated in Figure S1(ii), and their interactions result in the enhanced catenary optical field the gaps. Figure S1c,d show the magnitude profiles of $|E_x|$ and $|E_y|$ at the Ag-SiO_2 interface depicted in Figure S1(ii), respectively. The $|E_x|$ profile in the gaps can be well described by the well-known catenary curve in architecture (the fitting coefficients are greater than 0.998; the fitting catenaries are depicted in the form of black solid curves.). The generalized catenary model is given by:

$$|E_x| = a(\mathrm{e}^{bx} + \mathrm{e}^{-bx}) + c \tag{S1}$$

where a = 7.215e^{-3}, b = 6.934e^{7}, and c = 3.733. The catenary optical field will introduce an abrupt phase shift for the *x*-polarization incidence while it does not happen for the *y*-

polarization incidence. Shown in Figure S1e are the simulated reflective amplitudes of and phase difference between two orthonormal linearly polarized incidences along the *x*- and *y*-directions. The reflective amplitude of $|E_y|$ remains flat while the $|E_x|$ spectrum undergoes a dip around the resonant wavelength of 473 nm, owing to the absorption loss. Furthermore, the catenary optical field introduces an abrupt phase shift around 473 nm for the *x*-polarization, leading to a sharp propagation phase difference (close to 180°) between the *x*- and *y*-polarizations. As a result, narrowband photonic spin-orbit interactions will occur around this resonance wavelength.

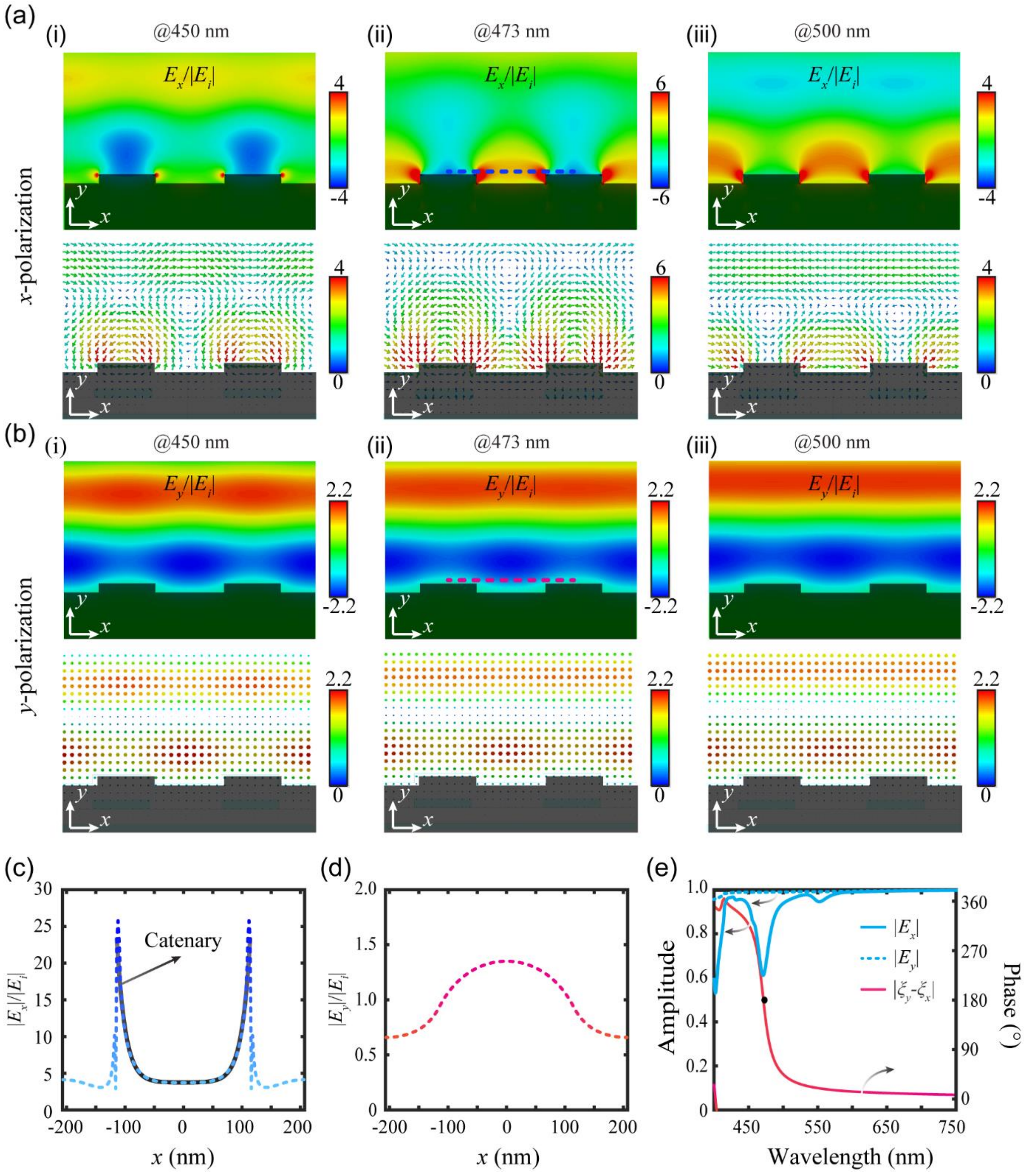

Figure S1. a,b) Simulated electric-field distributions of the blue PSG at different wavelengths under the illumination of a) *x*- and b) *y*-polarizations. Top row: 2D colour maps of real (E_x) / real (E_y) distributions. Bottom row: instantaneous electric-field lines. Extracted c) $|E_x|$ and d) $|E_y|$ profiles at Ag-SiO_2 interface shown in dash lines in Figure S1a(ii) and b(ii). The black solid curve in c) indicates fitting catenary curves. e) Simulated reflective amplitude spectra and phase difference for/between the *x*- and *y*-polarizations.

It is observed that the FWHM value of the red PSG is smaller than that of the blue one. To analyze its mechanism, the $|E_x|/|E_i|$ distributions of three PSGs were simulated at their peak

wavelengths under the illumination of the *x*-polarization light. As can be seen from Figure S2a-c, the red PSG (corresponding to the red color) can support stronger SPP compared with the blue one. Stronger the SPP is, sharper propagation phase difference is, as shown in Figure S2d. Essentially, the narrowband cross-polarized spectrum originates from the propagation phase difference between the *x*- and *y*-polarizations. As a result, the FWHM value of the red PSG is smaller than that of the blue one.

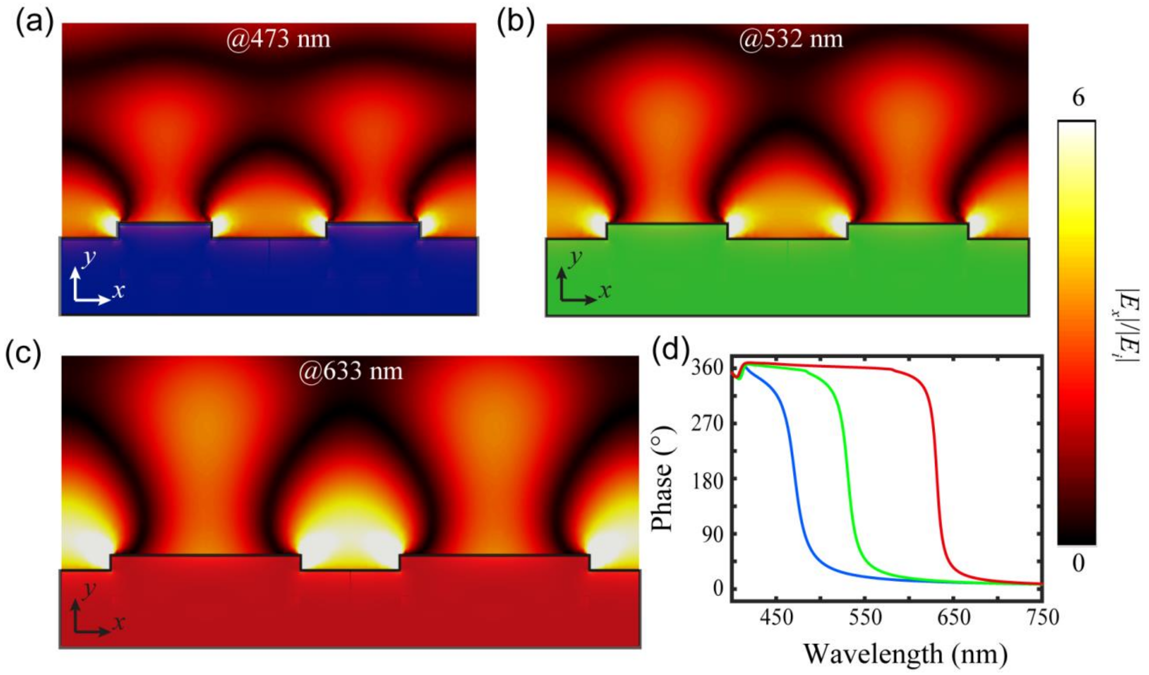

Figure S2. a-c) Simulated $|E_x|/|E_i|$ distributions of three PSGs at their peak wavelengths under the illumination of the *x*-polarization light. d) Simulated propagation phase difference between the *x*- and *y*-polarizations. Red, green, and blue curves respond to three PSGs, respectively.

2. Additional simulation and experimental results

Table S1. Comparisons between our work and several preliminary works on the dual-mode display.

Report [a)]	Color gamut in the print mode	Maximum FWHM	Hologram channels	SNR [b)]	Minimum peak efficiency	Lithography methods
Ref. [1]	Only two colors	>200 nm	One		~35%	Point-by-point
Ref. [2]	Only two colors	~80 nm	Two	~8:1	~18%	Point-by-point
Ref. [3]	About six colors	> 200 nm	One		<10%	Point-by-point
Ref. [4]	Only six colors	>200 nm	Three	~4:1	~66%	3D fabrication

Ref. [5]	Only five colors	>70 nm	Three	~4:1	~48%	3D fabrication
Ref. [6]	Full-color (~19.2%) c)	~75 nm	Three	~5:1	~34%	Point-by-point
Our work	Full-color (~38.1%) d)	~23.5 nm	Three	~17:1	~70%	Single-step

a) Ref. [1-6] correspond to the Ref. [25-30] in the main manuscript; b) Signal-to-noise ratio among several hologram wavelengths. c) 19.2% of the whole CIE 1931 chromaticity diagram; d) 38.1% of the whole CIE 1931 chromaticity diagram.

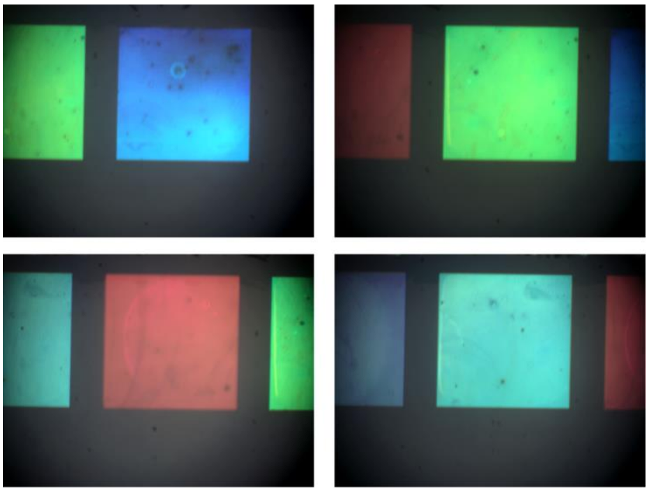

Figure S3. Measured optical microscope images showing that the area outside the PSGs is not black, which can prove the option that there is co-polarized CP component owing to the limited bandwidth performance of the elements in the decryption device (e.g., the quarter waveplate) and the ghost caused by multiple reflections among optical elements

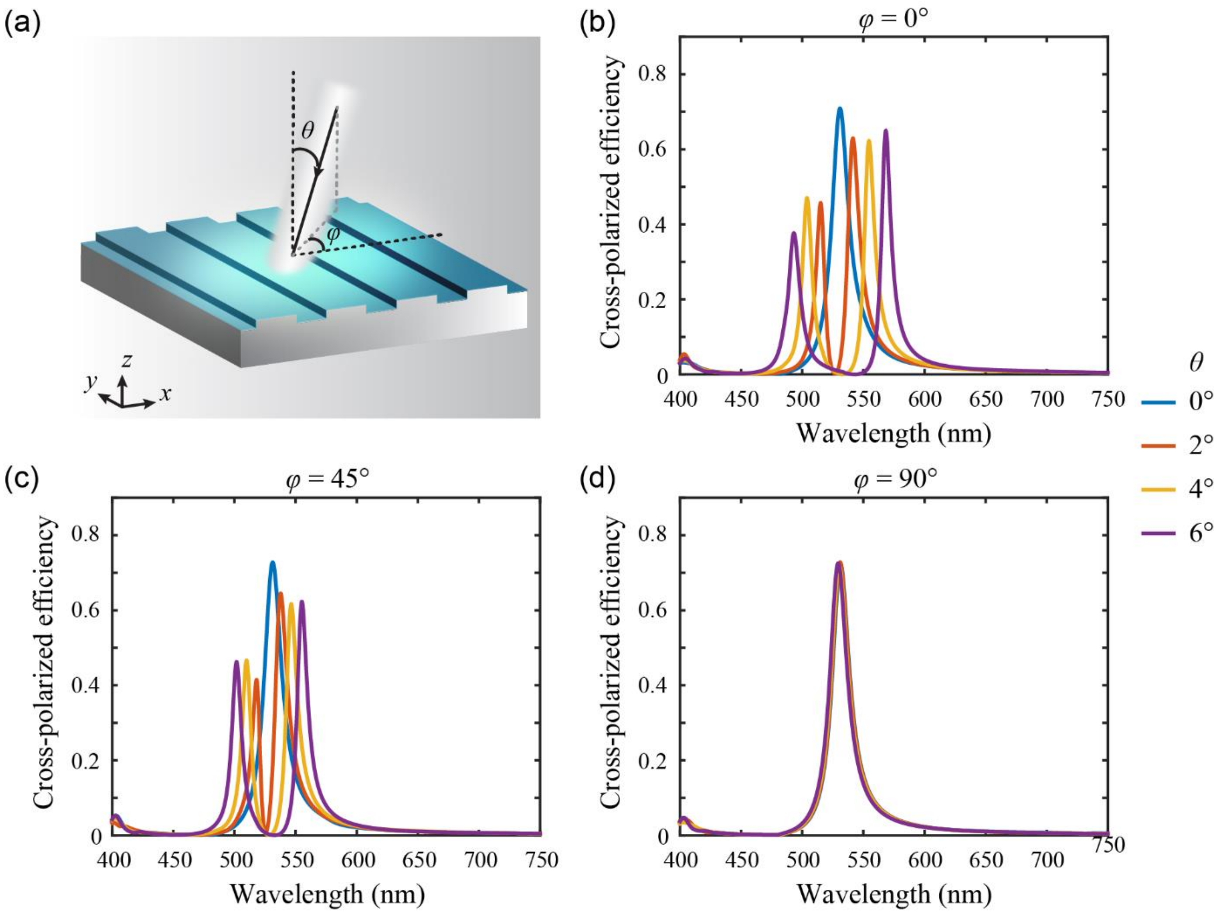

Figure S4. The verification of the fact that the none-normal illumination by a ×4/0.1 objective lens has a nonnegligible effect on the colours contributed by the cross-polarized CP component. a) The schematic illustration of oblique incidence. θ represents the angle between the normal line and incident direction, and φ indicates the corresponding azimuthal angle in the *xy*-plane. b-d) Simulated cross-polarized reflection spectra of the green PSG for different incident angles at different azimuthal angles. There are two resonances when obliquely illuminated for 0° and 45° azimuthal angles, and two peak wavelengths show two opposite shifts as illumination angle increases, which indicates that two SPP modes are coupled and contribute to sharp resonance for normal illumination. In contrast, the illumination angle has almost none effect on the spectrum when illuminated in the *yz*-plane.

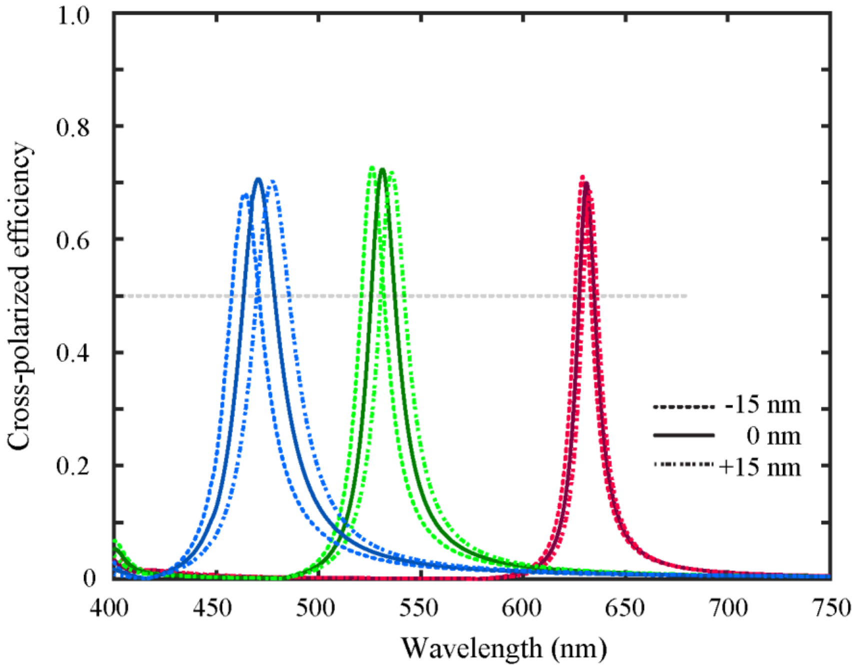

Figure S5. The simultated cross-polarized spectra of three PSGs with different fabrication errors for their widths. Within a fabrication tolerance of ~±15 nm, the maximum shift of the peak wavelengths is smaller than 7 nm, and the porization conversions are larger than 50% at three hologram wavelengths.

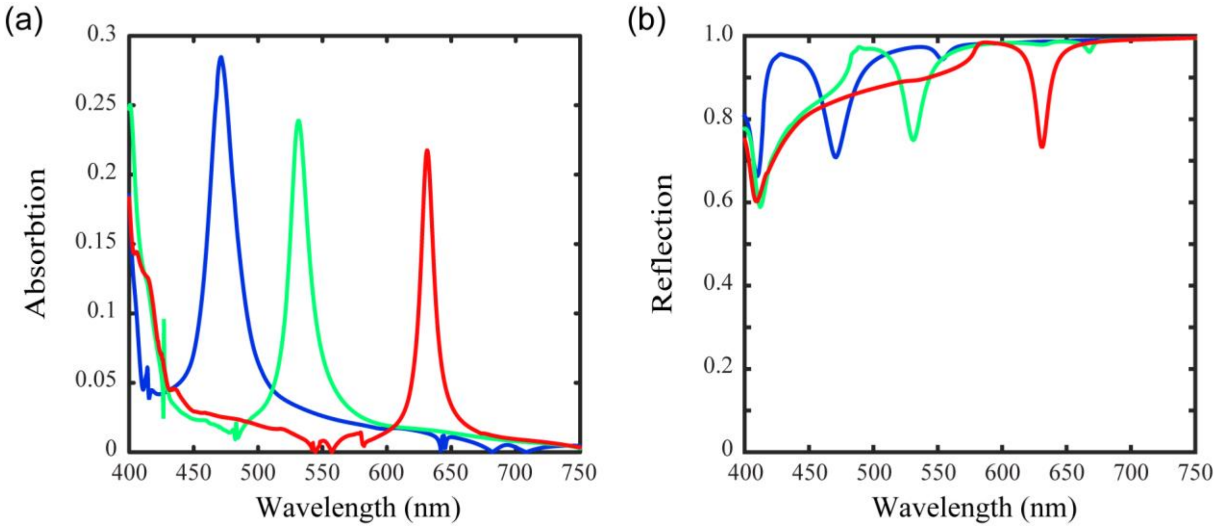

Figure S6. a) Simulated absorption spectra of three PSGs. b) Simulated reflection spectra of three PSGs.

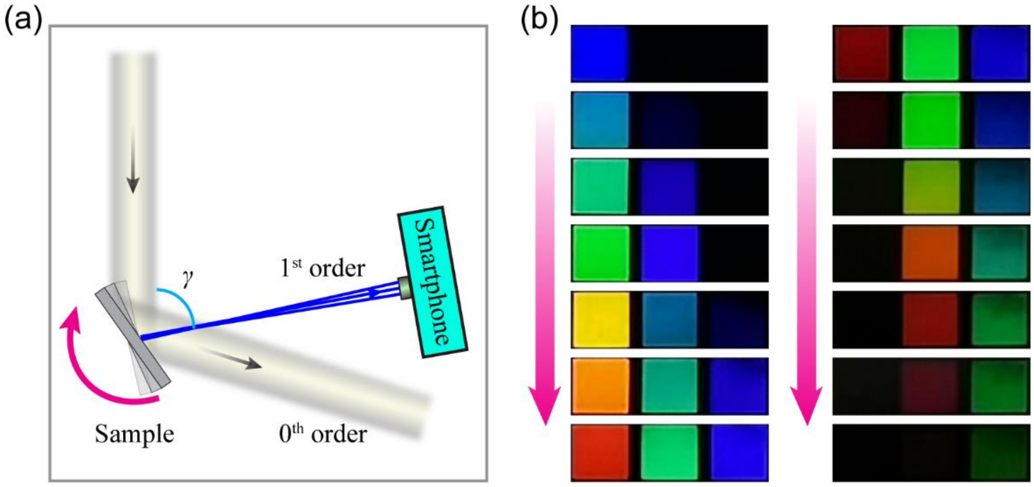

Figure S7. Experimental demonstration of high-purity colour observation for 1st-order diffraction light by a smartphone camera. a) The schematic illustration of the optical setup. The light is from the fluorescent lamp on the ceiling of the laboratory. The distance between the sample and light source/smartphone is approximately 2.5 m / 15 cm. Both smartphone and light source are fixed, but the sample is rotated. The angle γ between the incident and observation directions is about 70°. It is noteworthy that the grating line of PSGs is perpendicular to both illumination and observation planes. b) Measured optical images when the sample is rotated clockwise. The equivalent incident angle changes approximately from 60° to 88°.

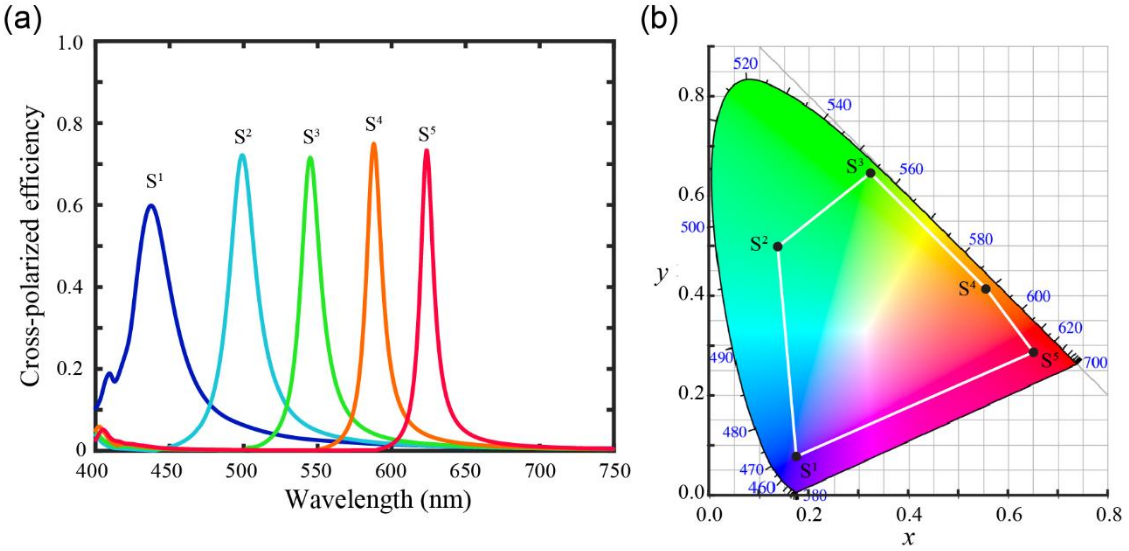

Figure S8. a) Simulated cross-polarized spectra of nine different PSGs. The periods of nine PSGs (from S^1 to S^5) are 320, 440, 500, 550, and 590 nm, respectively. The width is equal to half of period. b) The calculated the colors in the CIE 1931 chromaticity diagram from the cross-polarized spectra in Figure S8a. The color gamut is about 49.2% of the whole color gamut of the CIE 1931 chromaticity diagram.

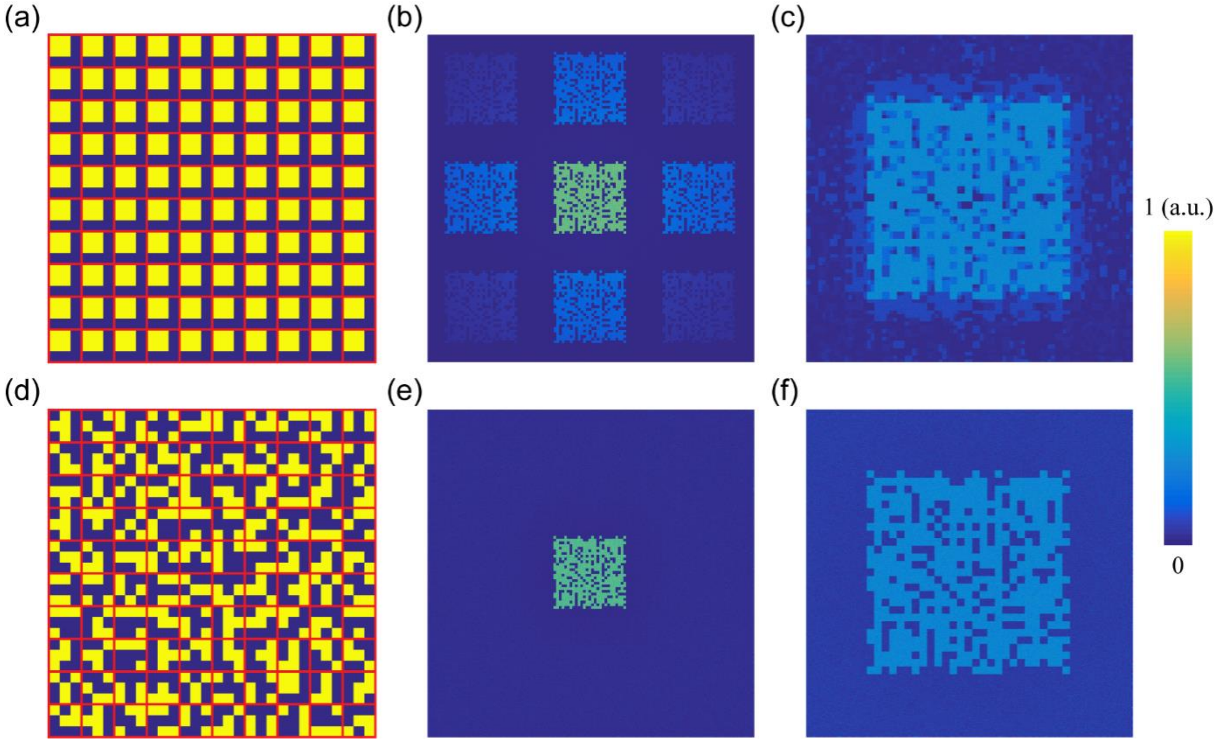

Figure S9. Simulated holographic images for periodic and random arrangement. For simplicity, we assume that the hologram displays one colour in the print mode and each supercell contains four subpixels. a-c) The periodic amplitude distribution and two simulated holographic images. There are oblivious high-order diffraction noises that will deteriorate the quality of the holographic image and reduce the available holographic space. d-f) The random amplitude distribution and two simulated holographic images, showing that the periodic noises are suppressed well.

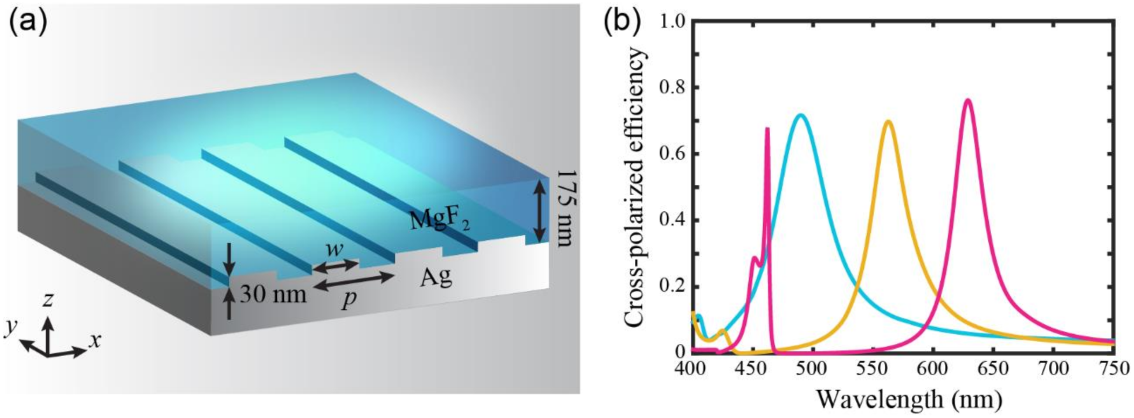

Figure S10. a) Simulated cross the schematic illustration of the PSG with a magnesium fluoride (MgF_2) covering film. b) Simulated cross-polarized spectra of three PSGs with the covering film shown in (a). Corresponding periods of cerulean, yellow, and curves are 180, 340, and 420 nm, respectively, and the width *w* is equal to half of the period. By further optimizing the width and period, the peak wavelength can be shifted toward either higher or lower wavelength.

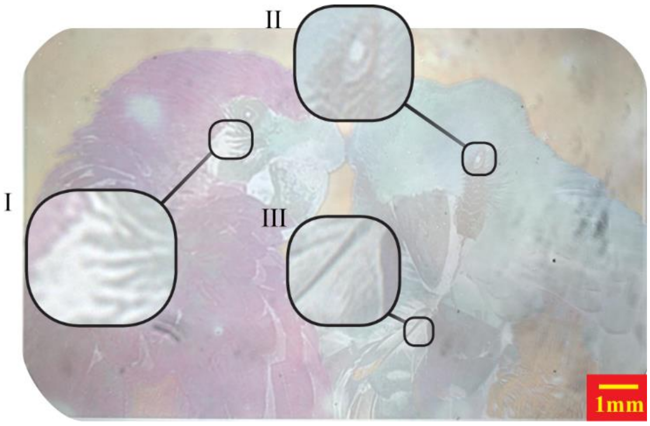

Figure S11. An optical image of the fabricated macaw meta-mark formed by a normal smartphone camera without the decryption device under the normal illumination of the light from a xenon lamp.

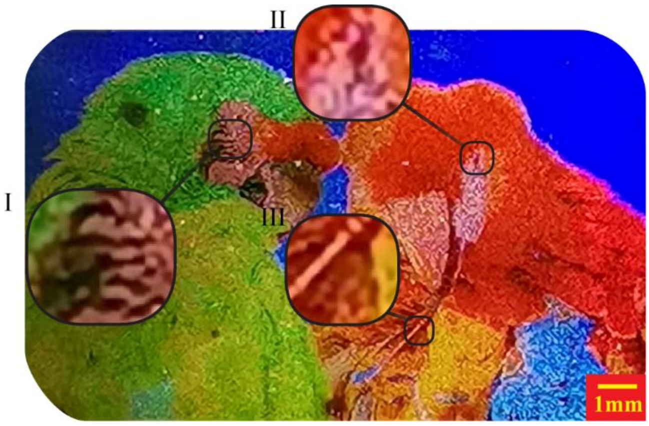

Figure S12. An optical image of the fabricated macaw meta-mark formed by a normal smartphone camera without the decryption device under the glance illumination of the light from a xenon lamp. The image quality is lower than the one contributed by direct reflect at normal incidence, because the 1st-order diffraction from PSGs whose grating lines are not perpendicular to the incident can be captured by the smartphone camera.

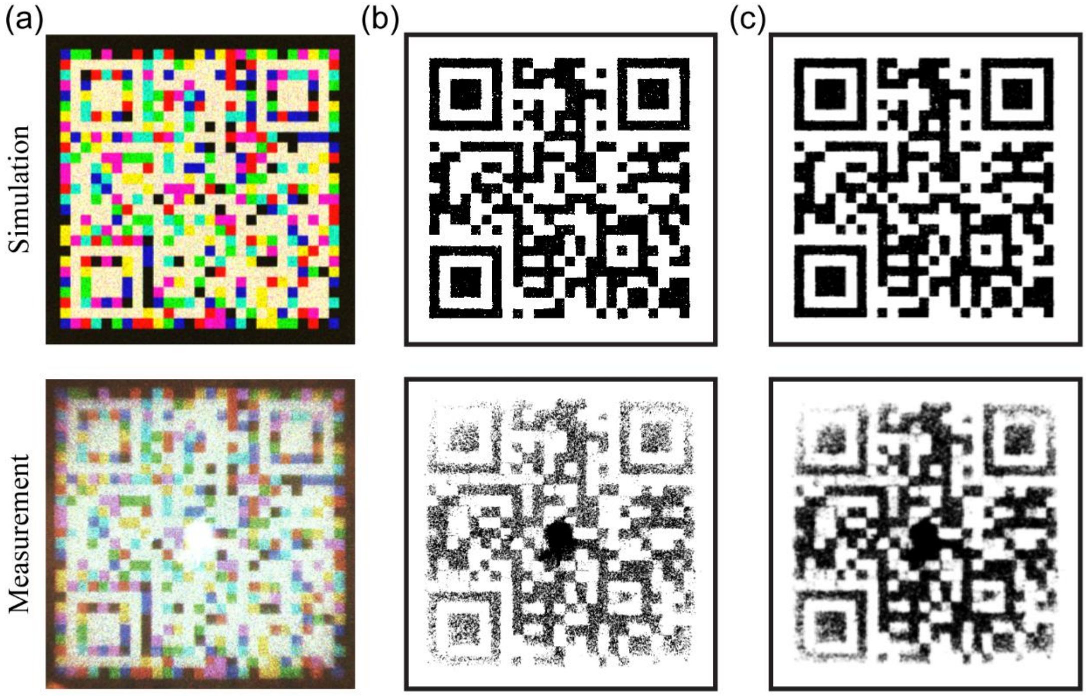

Figure S13. Simulated (top row) and measured (bottom row) results of the macaw meta-mark in the hologram mode. a) Synthetic holographic images at three wavelengths. b) Decrypted QR code patterns by extracting the effective part (the sum of RGB values is greater than 650) of the synthetic holographic images. c) Recognizable QR codes obtained by the convolution of Figure S13b that can reduce high-frequency noise.

3. Method

3.1 Simulations

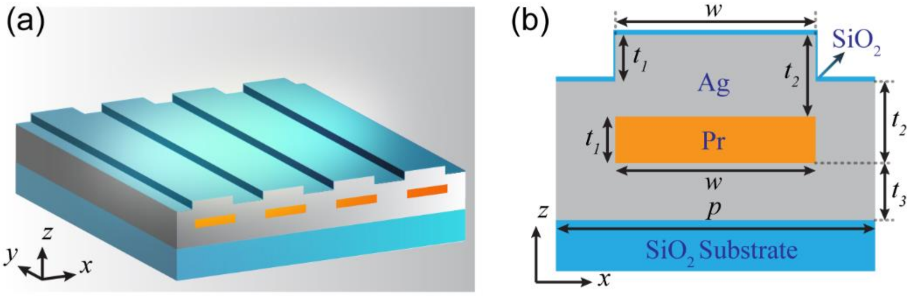

Figure S14. a) 3D and b) side views of the PSG in the simulations, showing the geometrical parameters with constant values of t_1 = 30 nm, t_2 = 80 nm, and t_3 = 50 nm. The thickness of the SiO_2 layer is 3 nm, but the side thickness is set as 1 nm considering the fabrication process. It is worth noting that the Pr pattern has little effect on the spectral response of the PSG. Pr: photoresist.

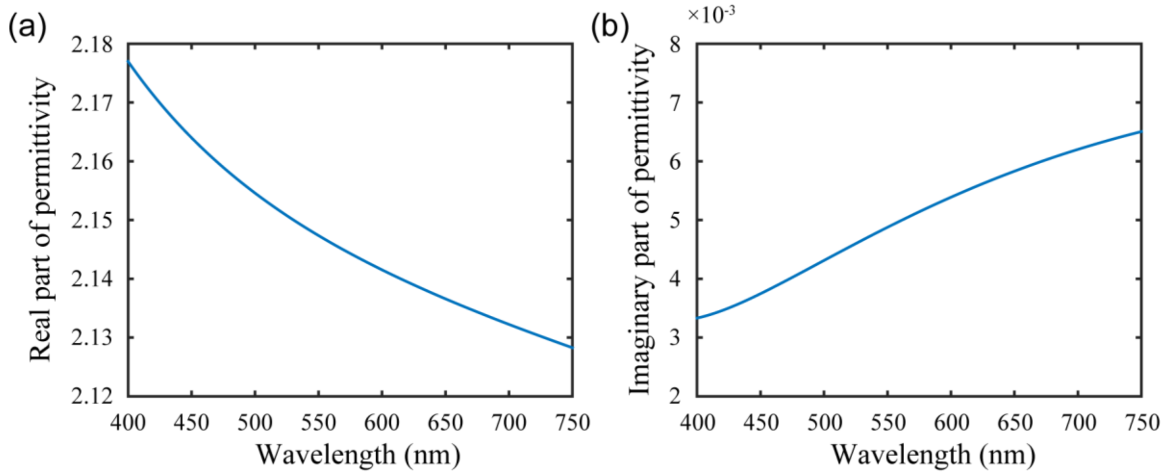

Figure S15. Measured a) real and b) imaginary parts of permittivity of SiO_2 after develop using SENTECH SE850.

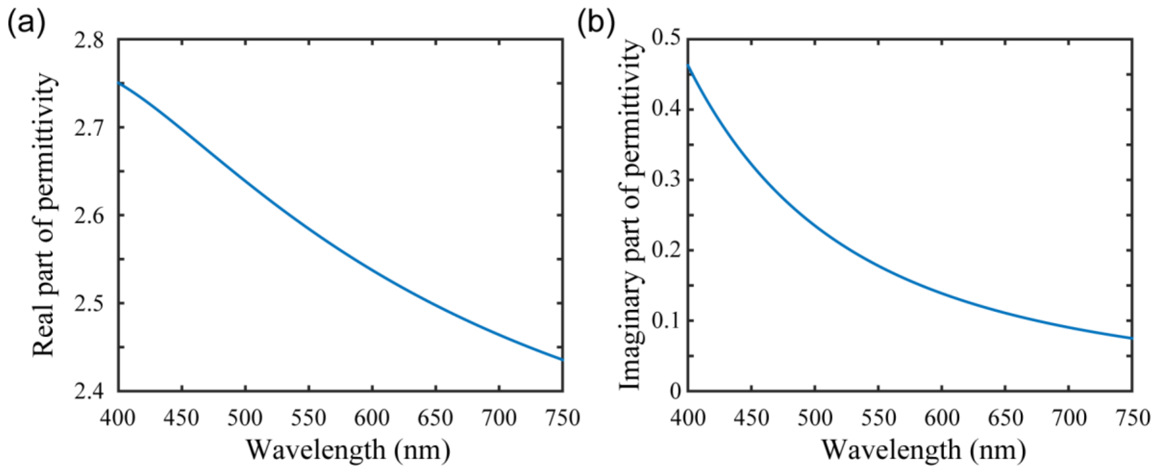

Figure S16. Measured a) real and b) imaginary parts of permittivity of photoresist using SENTECH SE850.

3.2. Experimental optical setups

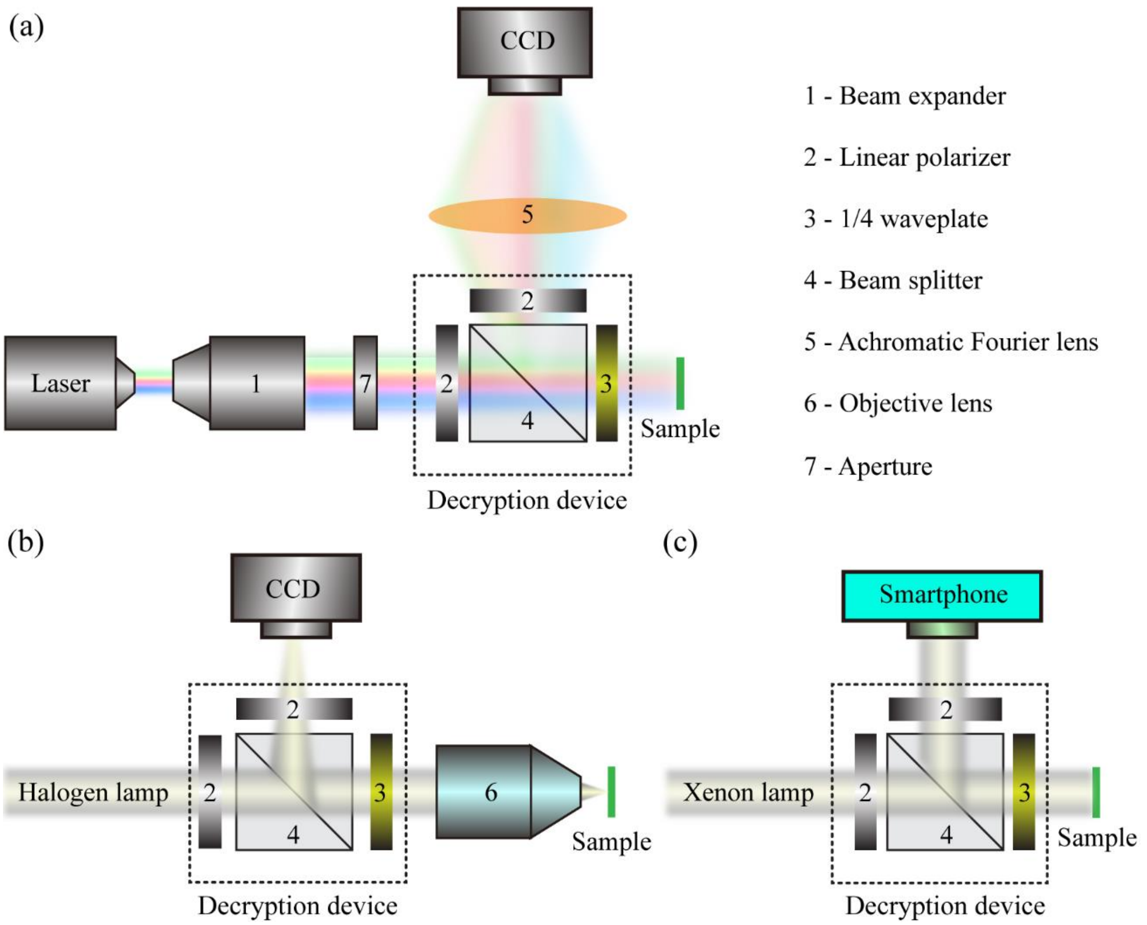

Figure S17. The schematic illustration of optical setups for the characterizations of fabricated samples. a) Characterizations of holographic images. b,c) Characterizations of optical images of the (b) landscape meta-mark, (c) macaw meta-mark, and PSG.

3.3. Modified Gerchberg-Saxton algorithm

Gerchberg-Saxton (GS) algorithm is widely applied to design phase-only holograms.[7] However, the far-field intensity profile obtained by the basic GS algorithm is far away from the ideal one and has a lot of white noise, which will deteriorate the quality of holographic image especially for multi-wavelength holography. To overcome this limitation, we present a modified GS algorithm that results in a near-optimum far-field intensity profile. The underlying idea of the new algorithm is to suppress the non-uniformity of the desired holographic region and to relax the noise suppression of the unused spatial-frequency space. As a result, the designed far-field intensity profile needs to be modified. For example, the image in the black box of Figure S18a is the target far-field intensity profile. Different from the basic GS algorithm, the intensity of the region outside the the black box is not equal to

zero but one (a.u.) for the new algorithm. Starting with a random initial phase distribution, the new algorithm is given as followed:

$$\begin{aligned} G_k(u) &= \mathcal{F}\left[g_k(x,y)\right] \\ &= \mathcal{F}\left\langle I_i(x,y)^{1/2} \times \exp\left\{i\phi[g_{k-1}(x,y)]\right\}\right\rangle \end{aligned} \tag{S2}$$

where k is the kth iteration; $\phi(*)$ is the argument of a complex function; F indicates Fourier transformation; g_k is the complex amplitude of the hologram; I_i is the input plane intensity profile. In order to suppress the non-uniformity of the desired holographic region and relax the noise suppression of the unused space, additional optimization steps are adopted:

$$C_k(v) = \left|G_{k\_N}(v)\right| \Big/ \left[I_o(v)^{1/2} + 1/\infty\right] \tag{S3}$$

$$w_k(v) = w_{k-1}(v) \times C_{k\_N}(v)^a \tag{S4}$$

where v is the concerned spatial-frequency space; G_{k_N} and C_{k_N} are the normalized G_k and C_k, respectively; I_o is the modified designed far-field intensity profile as mentioned above; w_0 = 1; a = 1. Then,

$$G_k^{'}(u) = I_o(u)^{1/2} \times w_k(u) \times \exp\left\{i\phi[G_k(u)]\right\} \tag{S5}$$

where u is the full spatial-frequency space. To suppress the singularities, they need to be forced down as followed:

$$c = b \times \sum\nolimits_v \left|G_k^{'}(v)\right| \Big/ L(v) \tag{S6}$$

$$G_k^{'}(\left|G_k^{'}(v)\right| > c) = c \tag{S7}$$

where b = 5 is an optimal valuel; $L(*)$ is the length of the set of number. Finally, the complex amplitude profile of the hologram is obtained by inverse Fourier transformation of the space-frequency profile as followed.

$$g_k^{'}(x,y) = \mathcal{F}^{-1}\left[G_k^{'}(u)\right] \tag{S8}$$

$$g_{k+1}(x,y) = I_i(x,y)^{1/2} \times \exp\left\{i\phi[g_k^{'}(x,y)]\right\} \tag{S9}$$

Figure S18b shows the far-field intensity distribution of output beam $|G_{50}(u)|^2$ after 50 iterations for I_i = 1. In contrast to the far-field intensity profile obtained by the basic GS algorithm (Figure S18c, also after also 50 iterations), the far-field intensity distribution in the concerned region obtained by the modified GS algorithm is very close to the ideal one with negligible noise. For the iteration process of the basic GS algorithm, the procedures of Equation S3, S4, S6 and S7 are removed.

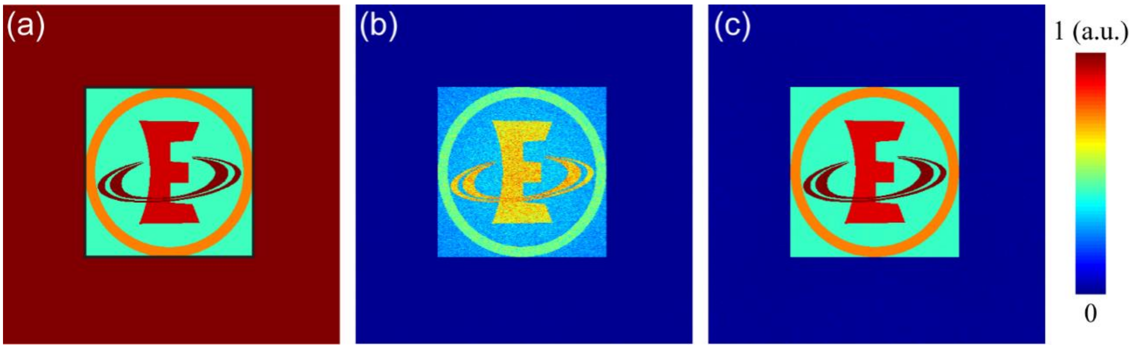

Figure S18. Comparisons of far-field intensity distributions obtained by basic and modified GS algorithms. a) Original image (just in the black box). b) Basic GS algorithm. c) Modified GS algorithm.

Reference

[1] G. Yoon, D. Lee, K. T. Nam, J. Rho, ACS Nano **2018**, *12*, 6421.

[2] Q. Wei, B. Sain, Y. Wang, B. Reineke, X. Li, L. Huang, T. Zentgraf, Nano Lett. **2019**, DOI: 10.1021/acs.nanolett.9b03957.

[3] Y. Zhang, L. Shi, D. Hu, S. Chen, S. Xie, Y. Lu, Y. Cao, Z. Zhu, L. Jin, B.-O. Guan, S. Rogge, X. Li, Nanoscale Horiz. **2019,** *4*, 601.

[4] K. T. P. Lim, H. Liu, Y. Liu, J. K. W. Yang, Nat. Commun. **2019**, *10*, 25.

[5] Y. Hu, X. Luo, Y. Chen, Q. Liu, X. Li, Y. Wang, N. Liu, H. Duan, Light Sci. Appl. **2019**, *8*, 86.

[6] Y. Bao, Y. Yu, H. Xu, C. Guo, J. Li, S. Sun, Z.-K. Zhou, C.-W. Qiu, X.-H. Wang, Light Sci. Appl. **2019**, *8*, 95.

[7] R. W. Gerchberg, *Optik* **1972**, *35*, 237.
